# Six-item cognitive impairment test (6-CIT)’s accuracy as a cognitive screening tool: best cut-off levels in emergency department setting

**DOI:** 10.3389/fmed.2023.1186502

**Published:** 2023-07-21

**Authors:** Francesco Salis, Daniela Pili, Manuel Collu, Luca Serchisu, Rosanna Laconi, Antonella Mandas

**Affiliations:** ^1^Department of Medical Sciences and Public Health, University of Cagliari, Cagliari, Italy; ^2^University Hospital “Azienda Ospedaliero-Universitaria” of Cagliari, Cagliari, Italy

**Keywords:** aging, cognitive impairment, screening, 6-item cognitive impairment test, mini mental state examination, emergency department

## Abstract

**Background:**

Nowadays, elderly patients represent a significant number of accesses to the Emergency Department (ED). Working rhythms do not allow to perform complete cognitive analysis, which would, however, be useful for the health care. This study aims to define the optimal cut-off values of the six-item Cognitive Impairment Test (6-CIT) as a cognitive screening tool in ED.

**Methods:**

This study included 215 subjects, evaluated at the Emergency Department of the University Hospital of Monserrato, Cagliari, Italy, from July to December 2021. The accuracy of 6-CIT as a cognitive screening tool was assessed by comparison with Mini Mental State Examination (MMSE).

**Results:**

The correlation coefficient between the two tests was −0.836 (CI: −0.87 to −0.79; *p* < 0.0001), and 6-CIT showed AUC = 0.947 (CI: 0.908–0.973; *p* < 0.0001). The 8/9 6-CIT cut-off score presented 86.76% sensitivity (CI: 76.4–93.8) and 91.84% specificity (CI: 86.2–95.7), and Youden index for this score was 0.786.

**Conclusion:**

Our study demonstrates that 6-CIT is a reliable cognitive screening tool in ED, offering excellent sensitivity and specificity with a 8/9 points cut-off score.

## Introduction

The elderly population represents a significant portion of the Emergency Department (ED) users (1, 2). Although EDs are often crowded, noisy places with reduced privacy—factors that can potentially affect performing cognitive tests—, early interception of cognitive disorders is essential to initiate timely treatment plans (3), including measures to prevent delirium, a burdened condition from a high mortality if not recognized and treated (4–6). The pain-related distress, the noise, and the unfamiliar environment have a profound effect on the patients, risking accentuating the symptoms (7), and a problem in clinical management and assistance to people with dementia is indeed represented by the impairment in the ability to report somatic symptoms: this ability seems to be directly linked to the insight of the disease (7, 8). ED staff are often not skilled to face the continuous—and progressively increasing over time—requests for care from the population affected by Neurocognitive Disorder, regardless of its severity (9).

Current models of emergency care do not adequately address the complex care needs of older patients, who have multiple—and often mutually related (10)—medical, functional, and social problems. Furthermore, the main geriatric problems are often neglected or not adequately considered, due to the unfamiliarity that the ED staff has with the management of these conditions: knowing how to recognize cognitive and sensory deficits, identify the functional state of the patient and the social resources at home are fundamental factors that guide both the diagnostic orientation and the therapeutic choices. In fact, in geriatric population, using an acute-pathology-focused method is limiting (11–14). A new care model has been proposed, the Geriatric Emergency Department, dedicated to this specific population, in which the classic color-coded *triage* is accompanied or replaced by tools dedicated to identifying the elderly high-risk patient (15–19). However, it is not possible to create dedicated facilities within each type of hospital. In low-volume hospitals, it would be sufficient to implement a series of measures to improve the management of the elderly patient: starting from staff training to ensure an immediate assessment of the patient’s cognitive and functional status, a systematic screening of conditions such as delirium (4), polypharmacy (20), and frailty (21), avoid hospitalization, where possible, and try to predict mortality (22–24). Effective management of the acute and chronic health problems of older people require models of care that emphasize continuity, completeness, and integration of services: these questions find an answer in Multidimensional Geriatric Assessment, usually applied in outpatient settings on patients suffering from several chronic diseases (25–29).

The abovementioned noise, overcrowding, and lacking time and privacy led us to assert that the administration, in EDs, of longer or harder to apply screening tests—such as Mini Mental State Examination, widespread in outpatient visits (28)—is almost unfeasible. But considering the need of early recognize cognitive deficits to better manage elderly patients, it would be unfair ignoring this aspect in ED visits. In 1999 a new quick instrument, called 6-item Cognitive Impairment Test (6-CIT), was validated (30) in community and outpatient setting, demonstrating good performances in the evaluation of cognitive impairment. Subsequently, other studies have confirmed their suitability in other settings and specific diseases (31–34). What is more, it was successfully tested to identify even mild cognitive impairment (35), which is a cognitive disorder which does not meet the clinical threshold for major neurocognitive disorder (36). Moreover, few studies focused on its reliability in detecting delirium, showing promising (37), yet not univocal evidence: in fact, it seems that it shows better performances if combined with other specific delirium-assessing tests (38, 39).

The aim of this study is to assess the applicability of 6-CIT as a first-level cognitive screening tool in the emergency department setting, identifying the cut-off score for optimal sensitivity and specificity by comparing it with MMSE.

## Methods

### Design of the study

This cross-sectional study included subjects evaluated at the Emergency Department of the University Hospital of Monserrato, Cagliari, Italy, from July to December 2021. The accuracy of 6-CIT as a cognitive screening tool was assessed by comparison with MMSE. INCLUSION CRITERIA: age ≥65 years; being subjected to 6-CIT and MMSE upon ED admission. EXCLUSION CRITERIA: age <65 years; inability to understand and/or speak Italian; presence of delirium; informed consent not provided. Two hundred and fifteen subjects met the inclusion criteria.

### Assessment

The enrolled subjects were evaluated for:

- 6-CIT (30), a simple first-level cognitive screening tool, composed of six questions: three about orientation [ask the patient what year (correct answer: 0 points; wrong answer: 4 points)—and month (0–3) we are in, and what time is it (0–3)], one about calculation [ask the patient to count backwards from 20 to 1 (correct answer: 0 points; 1 error: 2 points; >1 errors: 4 points)], one about attention [ask the patient to list the months of the year in reverse order (correct answer: 0 points; 1 error: 2 points; >1 errors: 4 points)], and one about delayed memory [ask the patient to repeat, at the end of the test, a 5-elements address (correct answer: 0 points; each error: 2 points, up to a maximum of 5 errors: 10 points)]. The sum scores from 0 (cognitively intact) to 28 (maximum impairment). In its validation, the 7/8 cut-off offered optimal sensitivity and specificity.
- Mini Mental State Examination (MMSE) (28, 40, 41), a widespread first-level cognitive screening tool, exploring orientation, memory, attention, language, and praxis. The sum of correct answers, corrected for age and education, scores from 30 (cognitively intact) to 0 (maximum impairment). A score <24 is suggestive of cognitive impairment.
- Activities of Daily Living (ADL) (42), used to assess the autonomy in performing common activities of everyday life, such as using the toilet, walking, or dressing up. The score ranges from 6 (completely autonomous) to 0 (completely dependent).
- Instrumental Activities of Daily Living (IADL) (42, 43) used to assess the autonomy in performing more complex tasks such as using the telephone or handling finances. The score ranges from 8 (completely autonomous) to 0 (completely dependent).
- For both ADL and IADL the information was collected with the help of the patients’ caregiver(s).
- Emergency Code (44), assigned during the triage: it can be red (emergency), yellow (urgent), green (delayed), white (expectant), or black (dead).
- Length of Stay (LOS) in the ED, measured in hours and minutes (hh:mm).

### Statistical analysis

Quantitative variables were expressed as frequencies, percentages, and Means ± Standard Deviations (SD), where appropriate; the variable “Age” was not-normally distributed, so it was backtransformed after logarithmic transformation and expressed as Mean. MMSE and 6-CIT scores’ correlation was studied with Pearson correlation coefficient (r). The analysis of variance was performed with ANOVA; Scheffé’s method was used for *post-hoc* analysis. 6-CIT performance was measured by Area Under *Receiver Operating Characteristic* (ROC) Curve (AUC). Youden’s J statistic was used to identify the optimal cut-off values. The multivariate analysis was performed with a logistic regression—stepwise, and variables with *p* > 0.1 were excluded by the model. Categorial variables were compared using the chi-squared test (χ^2^).

The results are reported indicating *p*-values in reference to 95% confidence intervals (C.I.).

MedCalc software (Version 19.5, Ostend, Belgium) was used for the statistical analysis.

## Results

The study included 215 subjects aged 65 years or more (mean: 78.1; SD: 6.97; ranged from 65 to 96), of whom 117 (54.4%) were women. Characteristics of the enrolled subjects are shown in Table 1.

**Table 1:** Characteristics of the sample.

Patients		Male	Female	
*n* (%)	215 (100%)	98 (45.6%)	117 (54.4%)	
Emergency code	White	Green	Yellow	Red
*n* (%)	1 (0.46%)	50 (23.26%)	161 (74.89%)	3 (1.39%)
Variables	MIN–MAX	Mean	SD	
Age (years)	65–96	78.1		
Education (years)	0–24	7.67	4.35	
6-CIT	0–28	7.43	7.3	
MMSE	0–30	24.57	5.58	
ADL	1–6	4.93	1.36	
IADL	0–8	5.39	2.67	
Length of stay in ED (hh:mm)	01:38–23:48	11:35	05:29	

6-CIT, six-item cognitive impairment test; MMSE, mini mental state examination; ADL, Activities of daily living; IADL, instrumental activities of daily living; ED, emergency department.

The emergency codes were distributed as follows: white (0.46%), green (23.26%), yellow (74.89%), and red (1.39%). Cognitive performances were explored with MMSE: according to the validation considering presence of cognitive deficit for <24 scores, 68 subjects (31.6%) were considered cognitively impaired. The evaluation of functional status was performed with ADL and IADL: 57.2% of the sample was considered dependent in ADL, and 66.9% was considered dependent in one or more IADLs. No gender difference has been found (*p* > 0.05). We stratified our population according to age in three groups: 65–74 years (34%), 75–84 years (49%), and ≥85 years (Table 2), finding a worsening of cognitive abilities (according to MMSE and 6-CIT) and functional abilities (ADL and IADL) with increasing age (*p* < 0.001).

**Table 2:** ANOVA and Scheffé *post-hoc*.

Variables	65–74 years Mean ± SD	75–84 years Mean ± SD	≥85 years Mean ± SD	ANOVA	Scheffé
6-CIT	4.27 ± 4.57	7.57 ± 7.14	13.71 ± 8.45	*p* < 0.001	1 vs. 2, 3 2 vs. 1, 3 3 vs. 1, 2
MMSE	26.25 ± 2.78	24.40 ± 5.02	20.46 ± 6.02	*p* < 0.001	1 vs. 2, 3 2 vs. 1, 3 3 vs. 1, 2
ADL	5.30 ± 1.14	4.97 ± 1.31	4.03 ± 1.54	*p* < 0.001	1 vs. 3 2 vs. 3 3 vs. 1, 2
IADL	6.57 ± 2.04	5.52 ± 2.48	2.54 ± 2.37	*p* < 0.001	1 vs. 2, 3 2 vs. 1, 3 3 vs. 1, 2

6-CIT, six-item cognitive impairment test; MMSE, mini mental state examination; ADL, activities of daily living; IADL, instrumental activities of daily living; SD, standard deviation.

**Figure 1:**
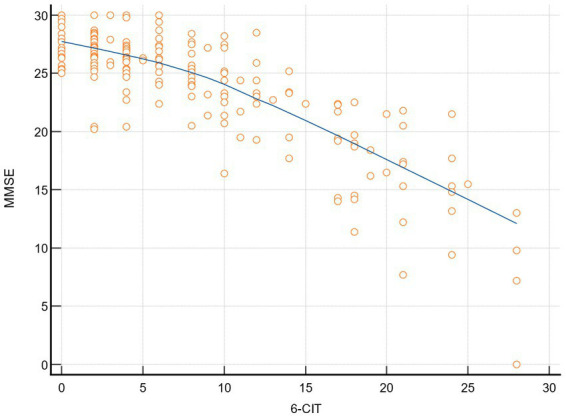
Correlation MMSE-6-CIT. 6-CIT, six-item cognitive impairment test; MMSE, mini mental state examination.

The correlation coefficient between the two cognitive screening tests—MMSE and 6-CIT—was −0.836 (CI: −0.87 to −0.79; *p* < 0.0001) (Figure 1).

**Figure 2:**
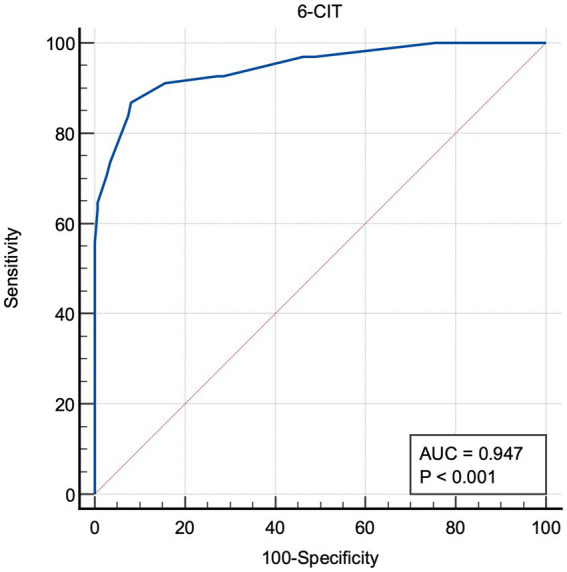
Area under the receiver operating characteristic curve (6-CIT). 6-CIT, six-item cognitive impairment test; AUC, area under the curve.

MMSE was then used as “classification variable” to screen people for cognitive deficit. 6-CIT had AUC = 0.947 (CI: 0.908–0.973; *p* < 0.0001) (Figure 2).

Sensitivity and Specificity for 6-CIT cut-off scores are shown in Table 3: the 5/6 cut-off score presents 92.65% sensitivity (CI: 83.7–97.6) and 72.79% specificity (CI: 64.8–79.8); the 6/7 presents 91.18% sensitivity (CI: 81.8–96.7) and 84.35% specificity (CI: 77.5–89.8); the 8/9 presents 86.76% sensitivity (CI: 76.4–93.8) and 91.84% specificity (CI: 86.2–95.7); the 9/10 presents 83.82% sensitivity (CI: 72.9–91.6) and 92.52% specificity (CI: 87.0–96.2).

**Table 3:** 6-CIT cut-off scores.

Cut-off score	Sensitivity (%)	95% CI	Specificity (%)	95% CI	+LR	−LR
0/1	100	94.7–100.0	24.49	17.8–32.3	1.32	0
2/3	97.06	89.8–99.6	51.02	42.7–59.3	1.98	0.058
3/4	97.06	89.8–99.6	53.74	45.3–62.0	2.1	0.055
4/5	92.65	83.7–97.6	71.43	63.4–78.6	3.24	0.1
5/6	92.65	83.7–97.6	72.79	64.8–79.8	3.4	0.1
6/7	91.18	81.8–96.7	84.35	77.5–89.8	5.83	0.1
8/9	86.76	76.4–93.8	91.84	86.2–95.7	10.63	0.14
9/10	83.82	72.9–91.6	92.52	87.0–96.2	11.2	0.17
10/11	73.53	61.4–83.5	96.6	92.2–98.9	21.62	0.27
11/12	70.59	58.3–81.0	97.28	93.2–99.3	25.94	0.3
12/13	64.71	52.2–75.9	99.32	96.3–100.0	95.12	0.36
13/14	63.24	50.7–74.6	99.32	96.3–100.0	92.96	0.37
14/15	55.88	43.3–67.9	100	97.5–100.0	0	0.44

CI, confidence interval; +LR, positive likelihood ratio; −LR, negative likelihood ratio.

Youden index was 0.786 for 8/9 cut-off score, which showed a positive likelihood ratio (+LR) of 10.63 and a negative likelihood ratio (−LR) of 0.14.

Then, we performed a logistic regression—stepwise, considering 6-CIT as dependent variable (we dichotomized it according to the 8/9 cut-off score), and age, gender, ADL, IADL, emergency code, and length of stay as independent variables. Female gender (OR: 0.4), and IADL (OR: 1.6) were positively independently associated with 6-CIT scores (AUC: 0.805, 95% C.I.: 0.745–0.855), while the others were non-significative regressors.

Finally, as in Table 4, we analyzed the reasons the subjects were admitted to the ED in light of cognitive impairment (according to the found threshold), and we did not find significant associations between them (χ^2^: 12.74, *p* = 0.8518).

**Table 4:** Reasons for admission.

	6-CIT	
Reason for admission	Absence of impairment	Cognitive impairment
Chest pain	10 (4.7%)	20 (9.3%)
Low back pain	1 (0.5%)	0 (0%)
Arryrhmia	3 (1.4%)	7 (3.3%)
Syncope/presyncope	2 (0.9%)	11 (5.1%)
Fatigue	0 (0%)	2 (0.9%)
Acute neurological injury	2 (0.9%)	2 (0.9%)
Vertigo	4 (1.9%)	9 (4.2%)
Other neurological cause	7 (3.3%)	8 (3.7%)
Cardiovascular cause	4 (1.9%)	9 (4.2%)
Nausea and/or vomiting	1 (0.5%)	3 (1.4%)
Anemia	3 (1.4%)	6 (2.8%)
Nonspecific pain	0 (0%)	1 (0.5%)
Trauma	5 (2.3%)	11 (5.1%)
Dyspnoea	7 (3.3%)	16 (7.4%)
Dermatological cause	2 (0.9%)	5 (2.3%)
Ophtalmologic cause	1 (0.5%)	4 (1.9%)
Abdominal pain	11 (5.1%)	26 (12.1%)
Fever	3 (1.4%)	3 (1.4%)
Acute kidney injury	1 (0.5%)	0 (0%)
Other	4 (1.9%)	3 (1.4%)

6-CIT, six-item cognitive impairment test.

## Discussion

The early assessment can help the management of the elderly patient (45) starting from the emergency department, up to the eventual hospitalization and subsequent discharge. Given the current state of affairs, as there are no standardized and universally recognized paths for the personalized management of these conditions (15–19), it is necessary that the emergency department staff have rapid and minimally invasive tools capable of intercepting the abovementioned deficits. Screening tests are more sensitive the more investigative, and therefore they cannot be routinely administered in the emergency room.

This reason led us to design this study, which aims to identify the optimal cut-off values of 6-CIT as a first-level cognitive screening tool in the emergency department. To pursue this objective, a sample of 215 subjects aged at least 65 was subjected to cognitive evaluation through 6-CIT and MMSE: the latter, born for outpatient setting, was used to discriminate patients with or without cognitive impairment. The two instruments showed a strong negative correlation (*r* = −0.836), with also an outstanding (46) AUC (0.947), considering MMSE as “classification variable,” as stated above. The cut-off scores that could express the best balance of sensitivity and specificity were then studied: in this sense, it was observed that the 8/9 cut-off score is able to optimally meet that demand, showing 86.76% sensitivity, and 91.84% specificity. Moreover, according to these scores, though analysis of variance seemed to suggest a worsening of cognitive capacity with increasing age, the multivariate model suggested that 6-CIT was not independently associated with age: this aspect confirms that cognitive impairment is not simply associated with aging, and that it should be framed into the complexity of geriatrics, which is not merely determined by the older age. Conversely, female gender was an independent regressor, consistently with the literature (47). Another consisting element, is that the mentioned worsening of cognitive capacity was independently related with compromission in autonomy of instrumental daily living (48). Also, for the sake of completeness, the absence of association with the LOS suffers from it being usually influenced by the availability of beds in the wards rather than a delay in the patients’ assessment.

Finally, we did not find any significant association between cognitive impairment and specific cause of access to ED, suggesting that patients suffering from different pathologies can benefit from a cognitive evaluation upon admission, whatever the complained symptoms.

Our study confirms that 6-CIT, is a reliable cognitive screening tool, which, being even simpler and quicker than MMSE, offers excellent sensitivity and specificity, as well as lending itself optimally to the needs of an emergency department. The study also shows that the optimal cut-off would be set for the score 8/9 points, differing from its validation (30). Knowing the best threshold could enable ED physicians to discriminate patients with and without cognitive impairment, thus allowing an actuation of personalized diagnostic and therapeutic protocols, as stated in the literature and in the best clinical practice, aiming to a more personalized approach (49–53). An early assessment and a patient-centered approach could contribute to achieve long-term outcomes for both patients and caregivers (54, 55), and even to enable patients to benefit from disease-modifying therapies (56).

Though of interest, the reported results show some limitations. First, our study is monocentric, and it could not represent all the Italian population: different regions and territories show educational and socioeconomical differences (57), which indeed are significant and independent determinants of cognitive impairment (58, 59). Second, most of the patients get green and yellow codes, so white and red codes are not widely represented: this aspect can likewise influence cognitive assessment, since most critical patients have more than a single cause to underperform during the stay in ED, from pain to lower hydration status, not to mention the pressure in clinical stabilization (60, 61). Third, we did not include patients gone to ED night-time, so our study does not consider whether this aspect could potentially influence the scores, as well as polypharmacy (20, 62, 63), which in turn can influence cognitive performances in elderly.

## Data availability statement

The original contributions presented in the study are included in the article/supplementary material, further inquiries can be directed to the corresponding author.

## Ethics statement

The studies involving human participants were reviewed and approved by Institutional Review Board of the University of Cagliari. Written informed consent for participation was not required for this study in accordance with the national legislation and the institutional requirements.

## Author contributions

FS, AM, MC, and RL contributed to the study design and the interpretation of the findings. DP, MC, and LS contributed to the data collection. FS, DP, and MC performed the statistical analysis. FS wrote the manuscript. All authors contributed to the article and approved the submitted version.

## Conflict of interest

The authors declare that the research was conducted in the absence of any commercial or financial relationships that could be construed as a potential conflict of interest.

## Publisher’s note

All claims expressed in this article are solely those of the authors and do not necessarily represent those of their affiliated organizations, or those of the publisher, the editors and the reviewers. Any product that may be evaluated in this article, or claim that may be made by its manufacturer, is not guaranteed or endorsed by the publisher.
